# Clinical presentation of congenital syphilis in a rotavirus vaccine cohort study in Lusaka: a case series 

**DOI:** 10.1186/s13256-021-02745-1

**Published:** 2021-04-01

**Authors:** Nsofwa Sukwa, Michelo Simuyandi, Masuzyo Chirwa, Yvonne Mutombo Kumwimba, Obvious N. Chilyabanyama, Natasha Laban, Aybüke Koyuncu, Roma Chilengi

**Affiliations:** Enteric Disease and Vaccines Research Unit (EDVRU), Centre for Infectious Disease Research in Zambia, Lusaka, Zambia

**Keywords:** Syphilis, Congenital syphilis, Infection, Case report, Infants, Zambia

## Abstract

**Background:**

Despite an otherwise robust national antenatal clinic program, maternal and congenital syphilis remains an important public health issue in Zambia. This case series reports the clinical presentation of seven infants diagnosed with congenital syphilis in Lusaka, Zambia.

**Case presentations:**

The cases in this series were incidental findings from a cohort of infants enrolled in a rotavirus vaccine immunogenicity study recruiting infants at 6 weeks of age. As part of clinical care for enrolled participants, we screened mothers of children who presented with adverse events of (i) repeated upper respiratory tract infections/coryza, (ii) skin lesions, and (iii) poor weight gain, for syphilis using rapid plasma reagin test. From a cohort of 214 mother–infant pairs enrolled between September and December 2018, a total of 115 (44.4%) of the mothers reported to have not been screened during antenatal care. Of these, four (3.5%) reported to have tested positive; and only two received treatment. Seven out of 57 (26.6%) children meeting the screening criteria had a positive rapid plasma reagin test result. The mean age at diagnosis was 4.5 months (1.3 months standard deviation), and the common presenting features included coryza (6/7), skin lesions (4/7), conjunctivitis (3/7), pallor/anemia (5/7), wasting (2/7), and underweight (5/7). Three of the seven infants were exposed to human immunodeficiency virus. Following diagnosis, all seven cases received standard treatment according to national treatment guidelines. That is, 6/7 cases received inpatient care with benzylpenicillin for 10 days, while 1/7 was treated as an outpatient and received daily procaine penicillin for 10 days.

**Conclusion:**

These findings suggest that, though screening for syphilis is part of the standard antenatal care in Zambia, it is not offered optimally. There is urgent need to address programmatic shortcomings in syphilis screening and treatment to avoid long-term sequelae. Additionally, clinicians need to raise their index of suspicion and rule out syphilis when confronted with these clinical symptoms, regardless of the mother’s human immunodeficiency virus status.

## Background

Syphilis is a sexually transmitted disease caused by *Treponema pallidum*, which, without appropriate antimicrobial therapy, results in chronic infection presenting with different clinical stages [1–7]. Many theories have been postulated as to its origins, including the pre-Columbian hypothesis, unitarian hypothesis, and Columbian hypothesis [8–10]. While the disease has for centuries been linked to sexual transmission, it was not until 1906 that the etiology was concretely identified and the pathogen classified as a bacterium *Treponema pallidum*. Later, in 1949, Nelson and Mayer described the *Treponema pallidum* immobilization test (TPI), the first truly specific serum reaction in the diagnosis of syphilis [11]. A radical change in treatment of syphilis occurred in 1943 following the discovery of penicillin, which to date remains the mainstay of treatment [11, 12]. Syphilis is divided into three well-characterized clinical stages, that is, primary, secondary, and tertiary, with primary syphilis presenting as a solitary, painless chancre. Without treatment, it develops into the secondary disease over weeks or months, manifesting in a range of clinical symptoms including fever, painless lymphadenopathy, diffuse rash, and genital or perineal condyloma latum. Beyond this comes the latent stage, during which the victim may be asymptomatic for a long time but remains positive for *T. pallidum* serologic tests. In the tertiary or late stage, the condition afflicts multiple organs, with complications in the nervous and cardiovascular systems [1, 7, 13, 14]. In pregnant women, transmission to the fetus mainly occurs *in utero* but also during birth, resulting in congenital syphilis. Syphilis can also cause adverse pregnancy outcomes such as preterm births, low birth weight, nonimmune hydrops fetalis, stillbirths, and perinatal death if not treated [6, 15–18]. Infants infected with syphilis are often asymptomatic at birth only to present with symptoms months to years later [19, 20]. Congenital syphilis is classified as either early congenital syphilis or late congenital syphilis when symptoms manifest before or after 2 years of age, respectively [20]. Symptoms of early congenital syphilis include rhinitis (“snuffles”), skin rash, hepatomegaly, splenomegaly, adenopathy, intrauterine growth restriction (IUGR), and anemia. Beyond 2 years of age, the clinical features become more distinct because of persistent inflammation and include dental and musculoskeletal manifestations such as Hutchinson’s teeth and saddle-nose deformity. The central nervous system is also often affected in late congenital syphilis, with patients presenting with conditions such as ocular syphilis (interstitial keratitis) and eighth-nerve deafness [19, 20].

The global burden of maternal syphilis remains high. In 2016, the estimated prevalence of maternal syphilis was 0.69% (95% confidence interval 0.57–0.81%) with a total of 988,000 case of syphilis in pregnant women [18]. This resulted in 661,000 (538,000–784,000) cases of congenital syphilis globally [18]. Without adequate treatment, congenital syphilis can lead to adverse birth outcomes including early fetal death, stillbirths, neonatal deaths, preterm births, and low birth weight. Of the 661,000 cases, an estimated 355,000 (290,000–419,000) cases had adverse birth outcomes [18], further making the case for wider screening and treatment coverage.

Because of its pathophysiology, syphilis also facilitates human immunodeficiency (HIV) infection in ways that make the two diseases huge public health problems; particularly in low–middle-income countries where the burden is greatest [21, 22]. Recent WHO guidance on the elimination of mother-to-child transmission of HIV and syphilis established targets of antenatal care coverage of 95%, and syphilis screening of 95%, to achieve a goal of fewer than 50 cases of congenital syphilis per 100,000 live births [23].

In Zambia, the burden of disease remains high. A national survey from 2016 found a 6.8% overall prevalence of syphilis among adults aged 15–59 years, with a prevalence of 7.2% among females and 6.3% among males [22]. This is similar to earlier findings reported in 2008 of a syphilis prevalence of 6.5% for women and 7.4% for men [24].

An earlier study looking at pregnant women admitted to the hospital whose pregnancies ended in either spontaneous abortion or stillbirth in 1982 found evidence of syphilis infection in up to 42% of women who aborted in the latter half of pregnancy [25]. Similarly, more recent studies have shown a continued high burden of maternal syphilis in Zambia despite the national antenatal program making HIV and syphilis testing mandatory during the first ANC visit [2, 26–29].

In this case series, we document the clinical presentation of infant diagnoses and management with congenital syphilis from a small cohort study on rotavirus vaccine immunogenicity from George Urban Health Centre in Lusaka, Zambia.

The seven cases presented in this series were drawn from a randomized-controlled trial of two versus three doses of Rotarix vaccine for boosting and longevity of vaccine immune responses in Zambia (ROVAS-2). Mother–infant pairs presenting to the George Maternal and Child Health department for scheduled 6-week immunizations were provided with study-specific information, and mothers interested in participating in the study were invited to the clinical research site. A total of 214 mother–infant pairs (infants aged between 6 and 12 weeks) were enrolled at the research site in George Health Clinic in Lusaka, Zambia from September 2018 to November 2019.

Sociodemographic data, water, sanitation, and hygiene (WASH) habits, as well as information regarding antenatal care such as attendance, screening for HIV/syphilis, and use of iron supplements, were collected during the screening and enrollment period. Of the 214 mothers who were enrolled in the study, 115 (53.7%) reported being screened for syphilis during routine antenatal care while 95 mothers (44.4%) were not screened for syphilis during the antenatal period. Mothers who were screened during antenatal care reported the results of the screening test, and it was found that 4/115 (3.5%) were RPR reactive, two of which (50%) received treatment for syphilis. Of the mothers who were screened, 111/115 (96.5%) were RPR nonreactive while four mothers (1.9%) reported not knowing the results of their RPR screening test.

**Fig. 1:**
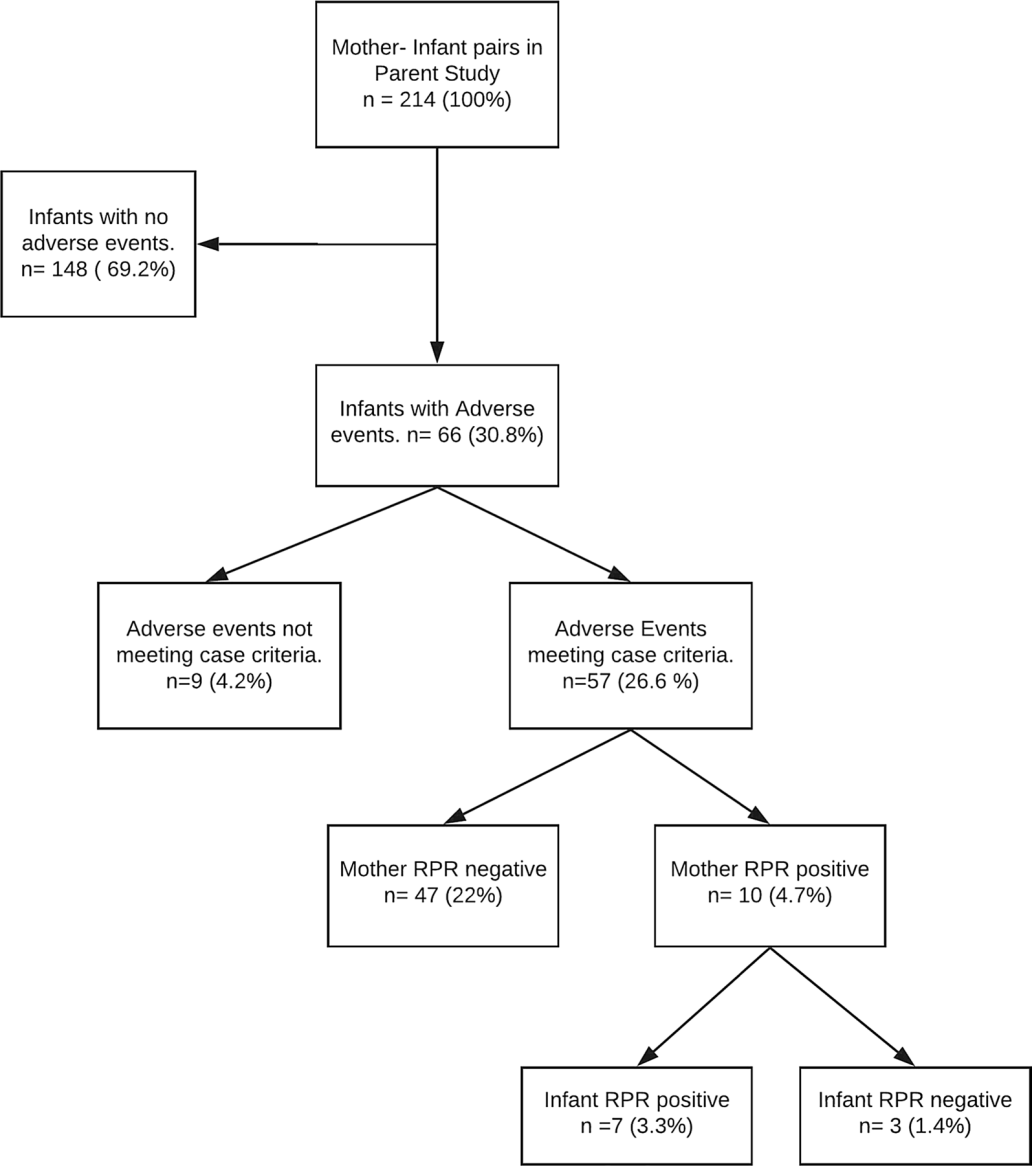
Study flow chart

As part of clinical care for enrolled participants, we screened 57 mothers of infants who presented with adverse events for syphilis using rapid plasma reagin (RPR) test. Mothers of infants were screened if the infant presented with (i) repeated upper respiratory tract infections/coryzal illness, (ii) skin lesions, and (iii) poor weight gain. Figure 1 shows the study flow of the participants in the case series.

All seven cases presented in this report were enrolled in the ROVAS 2 study at 6 weeks of age and were seen as part of routine clinical care at the George clinical research site while enrolled in the study. Specific characteristics of each case as follows:

## Case presentation 1

### Presentation at clinical research site

A 5-month-old female presented with a 3-day history of coughing and recurrent vaginal discharge and poor weight over the last 3 months. The discharge was said to have been present since birth, on and off, and it had worsened in the 3 days prior to presentation. Of note was that the child had been treated for conjunctivitis when she was 4 days old and for an upper respiratory tract infection (URTI) at 10 weeks of age. She was exclusively breastfed and, despite adequate milk supply, was small for her age. The mother was HIV negative and had not been screened for syphilis during pregnancy. There is a history of maternal febrile illness during pregnancy that was nonspecific.

### Medical examination and diagnosis

On examination, she was alert, pale, and small for her age (Wt 5.4 kg, length 64 cm, *Z* score: −3 SD to −2 SD). Vital signs were normal and, on respiratory examination, the child had bilateral crepitations. Abdominal and cardiovascular examinations were normal. Examination of the skin revealed generalized hyperpigmented spots (healing dermatitis). Genitourinary examination was also normal. RPR was reactive and with titers (1:4). FBC was done, and the patient was found to have microcytic hypochromic anemia (Hb 7.0 g/dL, MCV 59.7 fL, MCH 17.2 pg) and leukocytosis (WCC 15 × 10^3^/uL). Differential count was normal.

### Treatment and resolution

She was admitted and given aqueous crystalline penicillin G (benzylpenicillin) 270,000 IU given IV four times daily for 10 days. On follow up, respiratory and genitourinary symptoms had resolved**.**

## Case presentation 2

### Presentation at clinical research site

At 6 months of age, the child presented with a history of cough and fever for 1 day. The mother reported that he was treated for febrile illness of unknown cause at the George Health Centre OPD in the week prior to the current presentation. Past medical history revealed that that the child was HIV exposed and was admitted with a respiratory tract infection at 1 week of age. In addition, the child had been treated for dermatitis and an abscess at 3 and 4 months of age.

### Medical examination and diagnosis

The child was alert, pale, and not in respiratory distress but a whistling sound was noted on expiration. Oral thrush was also noted. Chest examination revealed bilateral crepitations. Other systems were unremarkable at time of examination. RPR was reactive, and FBC revealed microcytic hypochromic anemia (Hb 7.1 g/dL, MCV 71.9 fL, MCH 18.2 pg), lymphocytosis (72%), and monocytosis (11%).

### Treatment and resolution

He was admitted at Matero Level 1 Hospital and received benzylpenicillin 300,000 IU intravenously for 10 days. Following discharge, the mother returned to the facility and reported the child was much better and symptoms had resolved.

## Case presentation 3

### Presentation at clinical research site

At 6 months of age, the child and mother presented to the study clinic with a history of “peeling” skin rash on the palms and soles for the past 4 months. The rash was said to be intermittent and associated with recurrent episodes of cough and coryzal illness. The mother also reported noting a generalized rash during the week prior to presentation. Review of systems was nonrevealing. Of note, his medical history indicated that he was not HIV exposed and was delivered via spontaneous vaginal delivery at Matero Level 1 Hospital. Birth weight on record is 3.5 kg, and the mother reported that she was not screened for syphilis during antenatal care. The child had been treated for two episodes of acute diarrheal disease, both of which resolved uneventfully. The child was immunized according to Zambian national EPI guidelines.

### Medical examination and diagnosis

On examination by the study physician, the child was noted to be stable with snuffles and noisy breathing. His weight and height were recorded as 6.4 kg and 64 cm (− 1 SD to − 2 SD), respectively. Examination of the skin and subcutaneous system revealed multiple raised lesions over the chin as well as a desquamating rash over the child’s palms and soles. Abdominal examination revealed a palpable liver while his chest and cardiovascular examinations were normal. RPR and TPHA were both positive with titers of 1:32, and FBC showed microcytic, hypochromic anemia (Hb 10.2 g/dL, MCV 66.7 fL, MCH 21.3 pg) and leukocytosis of 17 × 10^3^/μL and lymphocytosis of 71%.

### Treatment and resolution

He was admitted to Matero Level 1 Hospital and treated with benzylpenicillin for 4 days and subsequent procaine penicillin 300,000 IU IM for 7 days. Upon completion of treatment, the mother reported that the child was much better and symptoms had resolved.

## Case presentation 4

### Presentation at clinical research site

This patient presented to the clinic with a history of cough and coryza at 5 months of age, which was treated as an URTI with normal saline drops. A month later, he presented with history of eye discharge and “sticky” eyelids for 3 days. On the basis of this presentation, the mother was screened for syphilis, and RPR test was reactive. Of note was that the child was born at term, with a birth weight of 3.7 kg and not exposed to HIV. There was no history of illness in the neonatal period. The child was exclusively breastfed and was growing and developing as expected.

### Medical examination and diagnosis

On examination, the child was alert and active and appeared well nourished (Wt 6.8 kg, length 67 cm, *Z* score −2 SD to −1 SD). Vital signs were normal. Local examination of the eyes revealed no obvious eye discharge. Respiratory, abdominal, and cardiovascular examinations were normal. RPR was reactive, but titers were not done. FBC was done, and the patient was found to have normocytic hypochromic anemia (Hb 9.1 g/dL, MCV 72.9 fL, MCH 22.9 pg). Differential count showed lymphocytosis.

### Treatment and resolution

He was admitted as a case of asymptomatic congenital syphilis and given aqueous crystalline penicillin G (benzylpenicillin) 355,000 IU IV four times daily for 10 days. Following treatment with intravenous antibiotics, all symptoms resolved.

## Case presentation 5

### Presentation at clinical research site

This patient presented at 4 months of age with coughing and sneezing for 2 days. She was treated for coryzal illness with normal saline nasal drops. At the time, the patient’s mother was screened for syphilis on the basis of screening criteria and RPR test was positive. Of note in her past medical history, the child was HIV exposed and the mother has a history of nonspecific rash around the neck during the neonatal period as well as peeling of skin on the plantar and palmar surfaces of the feet and hands, respectively, soon after birth. Birth history revealed that she was born at term with a birth weight of 3.4 kg.

### Medical examination and diagnosis

On examination, she was alert, active, and not in respiratory distress. She was not pale or jaundiced. All vital signs were within normal range. She appeared small for her age (Wt 4.6 kg, Ht 58.5 cm, *Z* score: −3 < −2). Chest examination was normal.

### Treatment and resolution

Because of domestic constraints, she was treated as an outpatient with procaine penicillin G 230,000 IU given IM once daily for 10 days, after which all symptoms resolved.

## Case presentation 6

At 4 months of age, the participant presented with a 1-week history of diarrhea and cough with associated history of eye discharge and fever for 3 days. The child was reported to be active and breastfeeding well. She was born at home via spontaneous vaginal delivery and was admitted with sepsis soon after birth at the tertiary hospital. The birth weight on record is 3.0 kg, and the child was not HIV exposed.

### Medical examination and diagnosis

On examination, the child was noted to be stable and afebrile and was neither pale nor jaundiced. She was not in respiratory distress and was well hydrated. Her weight was 5.6 kg and her height 64 cm, with a temperature of 37.6 °C. A diagnosis of acute diarrheal disease with coryzal illness was made, but congenital syphilis still needed to be ruled out. RPR test was ordered for both the mother and infant and both were positive.

### Treatment and resolution

A diagnosis of congenital syphilis was made, and the child was referred to Matero Level 1 Hospital**.** The child was admitted and received benzylpenicillin 300,000 IU intravenously four times daily for 10 days. The mother returned to the study site and reported that all symptoms had resolved.

## Case presentation 7

### Presentation at clinical research site

A male infant, 2 months old, presented with a history of breathing difficulty, coryza, and cough for 1 week, with symptoms becoming progressively worse in the last 2 days

### Medical examination and diagnosis

On examination, the child appeared ill and tachypneic, and was neither pale nor jaundiced. The child also had oral candidiasis and inguinal lymphadenopathy. His weight and length were 3.8 kg and 53 cm, respectively, with an axillary temperature of 36.6 ℃. Examination of the respiratory system revealed flaring of nostrils, as well as subcostal and intercostal recessions with bilateral crepitations. Other systems were normal.

### Treatment and resolution

The child was diagnosed with pneumonia with RVD exposure, and he was admitted for further management including administration of intravenous benzylpenicillin 6 hourly, oxygen by nasal prongs, and suctioning of oral and nasal secretions. RPR test was done on both the mother and infant and was found to be positive. The child was admitted to the Children’s Hospital at the UTH for 10 days, and on follow-up visits the child was well and symptoms had resolved.

Among the seven confirmed cases of congenital syphilis, coryzal symptoms were the most common and were observed in 6/7 cases. Skin lesions and anemia were present in 4/7 cases while conjunctivitis occurred in 3/7 (Table 1).

**Table 1:** Summary of clinical characteristics of the seven cases

Clinical characteristics	Case 1	Case 2	Case 3	Case 4	Case 5	Case 6	Case 7
Coryza/URTI	+	+	+	+	−	+	+
Skin lesion	+	+	+	−	+	−	−
Conjunctivitis	+	−	−	+	−	+	−
Musculoskeletal deformities	−	−	−	−	−	−	−
Vaginal discharge	+	N/A	N/A	N/A	−	−	NA
Hepatomegaly	−	−	+	−	−	−	−
Preterm birth	+	−	−	−	−	−	−
Anemia/pallor	+	+	+	−	+	-	+

+, present; −, absent; N/A, not applicable; URTI, upper respiratory tract infection

Clinical characteristics of the case studies are described in Table 2. Symptoms involving the upper respiratory tract were the most common presenting symptom, and the age at diagnosis ranged from 30–180 days  (median = 42 days, interquartile range (IQR) 5–105 days).

**Table 2:** Summary of clinical and laboratory investigations done

Characteristic	Case 1	Case 2	Case 3	Case 4	Case 5	Case 6	Case 7	Mean (SD)
Age at presentation (days)	4	5	42	120	28	105	60	Median (IQR) 42 (5–105)
Age at diagnosis (months)	5	5.5	6	5	4	4	2	4.5 (1.3)
Presenting symptoms	Conjunctivitis	RTI	Coryza	RTI	Dermatitis	Coryza	Coryza	
Birth weight (kg)	3.2	2.2	3.5	3.7	3.4	3.6	2.9	3.2 (0.5)
Mother screened at antenatal care	No	No	No	Yes (NR)	No	No	No	
HIV status	CNE	CE	CNE	CNE	CE	CNE	CE	
Weight at diagnosis (kg)	5.4	6.3	6.4	6.8	4.6	5.6	3.8	5.6 (1.1)
Length at diagnosis (cm)	64	63	64	67	58.5	64	53	61.9 (4.7)
*Z*-score at diagnosis								
Weight for length *z* score (WHZ) (wasting < −2 SD)	−3 < −2	−2 < −1	−2 < −1	−2 < −1	−2	−3 < −2	−1 < Med	
Wt for age *z* score (WAZ) (under Wt = < −2 SD)	−2 SD	−2 SD < −1 SD	−2 SD	−1 < Median	−3 < −2	−3 < −2	−3	
TPHA titers (1:8)	1:4	–	1:32	–	–	–	–	
FBC								
Hemoglobin (10.5–14 g/dL)	7.0	7.1	10.2	9.1			8.2	8.3 (1.4)
MCV (72–88 fL)	59.7	71.9	66.7	72.9			85.3	71.3 (9.4)
MCH (24–30 pg)	17.2	18.2	21.3	22.9			26.2	21.2 (3.6)
WCC (6–14 × 10^3^/μL)	15	10.4	17	14			16.79	14.6 (2.7)
Platelets (150–450 × 10^3^/μL)	300	594	657	344			255	430 (182.6)
Differential count								
Neutrophils (16–52%)	31	15	23	16			27.4	22.5 (7)
Lymphocytes (32–69%)	64	72	71	79			55.8	68.4 (8.8)
Monocytes (2–8%)	2	11	4	3			16.2	7.2 (6.1)
Eosinophils (0–5%)	3	1	2	2			0.4	1.7 (1)
Basophils (0–2%)	0	1	0	0			0.2	0.2 (0.4)

CNE, no perinatal HIV exposure—child not exposed; CE, perinatal HIV exposure—child exposed; NR, nonreactive; RTI, respiratory tract infection; SD, standard deviation; TPHA, *Treponema pallidum* hemagglutination assay

Three of the infants were HIV exposed, and there was only one case in which the mother had been screened for syphilis during antenatal care with the self-reported result being negative.

Regarding the nutritional status of the cases, three of the children were wasted (≤ 2 SD WHZ) and underweight (≤ 2 SD WAZ) at the time of diagnosis, and in all the five cases where full blood count was done the children were found to be anemic (microcytic hypochromic anemia) with a raised total white cell count. Of the two cases where TPHA titers were measured, only one had titers above 1:8, which is the point at which it is a true reactive test.

## Discussion

This case series report documents an incidental finding of congenital syphilis in a cohort study involving infants aged 6 weeks, aimed at evaluating the potential boosting effect of a third dose of Rotarix. These cases presented as adverse events (AEs) during the follow-up period of the study.

We reported a wide age range at first presentation (that is, 4 days to 4 months), consistent with literature, which states that about 60% infants infected with syphilis congenitally are asymptomatic at birth [19].

A second finding is that the average age at diagnosis was around 5 months. Although these children presented to the health facility at a younger age, they were only diagnosed at a much later stage of the disease because of a low index of suspicion on the part of the health worker. The clinical presentation of congenital syphilis is often nonspecific in nature [19], and clinicians often miss the diagnosis because of its subtle presentation. Notably, five out of seven infants (over 70%) were underweight or presented with poor weight gain. The fact that the slow growth trajectory of these infants did not trigger an investigation until 5 months is also indicative of the low index of suspicion for syphilis.

Given that syphilis infection often facilitates the transmission of HIV [21, 30], it was unsurprising to find that 3/7 (42.8%) of the case infants were exposed to HIV; this implies that we need to continue to screen pregnant women for syphilis irrespective of HIV status.

While these data are observational in a small cohort of infants, we believe this could reflect a broader problem nationally, if not regionally. Thus, there is need to increase access to comprehensive antenatal care overall to increase the number of pregnant women who undergo early screening and treatment for both HIV and syphilis [18, 31]. From a health system perspective, the fact that just over half (53%) of the women reported to have been screened for syphilis, and that out of those who were positive only half received treatment, highlights the gaps in the system. RPR screening is supposed to be mandatory in antenatal clinics, and determining the factors contributing to the low uptake or offer of the service is important [2, 27, 29]. All but one of the cases come from mothers who were not screened antenatally. Our results suggest that Zambia may be behind the WHO global targets for ANC care coverage of 95%, and syphilis screening of 95%. This must be improved to achieve a goal of fewer than 50 cases of congenital syphilis. Although limited by sample size, our data suggest that there are active infections among pregnant mothers that are not detected and consequently remain untreated. Further, it is likely that their sexual partners also remain infected and untreated. Our findings have huge implications also for HIV prevention and control programs, given that sexually transmitted infections have been shown to be positively associated with HIV infection [3, 32, 33].

Lastly, congenital syphilis can potentially lead to severe long-term sequelae in infected infants if left untreated [20, 23]. Despite generally normal birth weight among our cases, several infants were already underweight and wasted at diagnosis [weight for age z score (WAZ) ranged between median to −3 SD, and height for age *z* score (HAZ) ranged between median and −3 SD]. It is apparent that syphilis was already taking its toll among the cases. Malnutrition triggers a cascade of other dysfunctions and increases the risk of other opportunistic infections, adding to the burden of morbidity in the infected child [34, 35].

To attain the target of reducing mother-to-child transmission of HIV and syphilis to below 50/100,000 live births [18], we propose the following steps.

- Data on antenatal programs in Zambia need to be collected systematically to document the successes and failures of the program adequately. This will, in turn, inform interventions that will improve screening and treatment of syphilis during routine antenatal care.
- Combined HIV/syphilis rapid diagnostic tests (RDTs) need to be introduced to match the current success of the PTMCT program.
- Spousal/partner disclosure should be encouraged, the lack of which can be a major barrier to treatment despite timely diagnosis.
- Clinicians need to raise their index of suspicion and rule out congenital syphilis given its nonspecific and often subtle presentation.

## Conclusion

The clinical presentation of congenital syphilis is often subtle and nonspecific, which can lead to delays in diagnosis and treatment of cases. Therefore, clinicians need to raise their index of suspicion and rule out syphilis when confronted with these clinical symptoms in both HIV-exposed and non-exposed infants. Robust screening of pregnant women during antenatal care is an effective way to reduce maternal syphilis and subsequent congenital transmission. These findings suggest that, although screening for syphilis is part of the standard antenatal in Zambia, it is not offered optimally. Hence, there is urgent need to address programmatic shortcomings in maternal syphilis screening and treatment to avoid long-term sequelae in infants.

## Data Availability

The authors declare that the data and materials used in this study are the property of the Centre for Infectious Disease Research in Zambia (CIDRZ), and it is their responsibility to make it available on request.

## Abbreviations

RPR: Rapid plasma reagin test
RDT: Rapid diagnostic test
RVD: Retroviral disease
Wt: Weight
Ht: Height
IU: International units
CNE: Child not exposed (no perinatal HIV exposure)
CE: Child exposed (perinatal HIV exposure)
NR: Nonreactive
RTI: Respiratory tract infection
SD: Standard deviation
OPD: Outpatient department
EPI: Expanded program on immunization
FBC/DC: Full blood count/differential count.
Hb: Hemoglobin
MCV: Mean corpuscular volume
MCH: Mean corpuscular hemoglobin
WCC: White cell count
IV: Intravenously
IM: Intramuscular
ANC: Antenatal clinic
UNZABREC: University of Zambia Biomedical Research Ethics Committee
NHRA: National Health Research Authority
ZAMRA: Zambia Medicines Regulatory Authority
EDCTP: European and Developing Countries Clinical trials Partnership
CIDRZ: Centre for Infectious Disease Research in Zambia
TPHA: *Treponema pallidum* hemagglutination assay
WHO: World Health Organization
URTI: Upper respiratory tract infection
